# 2-Eth­oxy-6-[(methyl­imino)­meth­yl]phenol

**DOI:** 10.1107/S1600536810019951

**Published:** 2010-06-05

**Authors:** Cheng Min Ge, Shu-Hua Zhang, Feng Chao, Yin Guang Wang, Wei Li

**Affiliations:** a College of Chemistry and Bioengineering, Guilin University of Technology, Guilin 541004, People’s Republic of China

## Abstract

In the title compound, C_10_H_13_NO_2_, synthesized by the reaction of 2-hy­droxy-3-eth­oxy­benzaldehyde with methyl­amine, there is an an intra­molecular O—H⋯N hydrogen bond involving the hy­droxy substituent and the amino N atom. In the crystal, mol­ecules form inversion dimers connected by pairs of C—H⋯O hydrogen bonds.

## Related literature

For similar Schiff bases, see: Chatziefthimiou *et al.* (2006 ▶); Zhang *et al.* (2003 ▶); Kargar *et al.* (2010 ▶). For related structures, see: Karadayı *et al.* (2003 ▶); Che *et al.* (2002 ▶); Jia *et al.* (2009 ▶); Fun *et al.* (2009 ▶). For structures with similar hydrogen-bonding to the title compound, see: Wang *et al.* (2010 ▶); Kargar *et al.* (2010 ▶). 
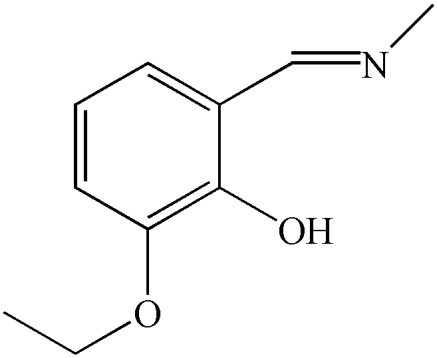


## Experimental

### 

#### Crystal data

C_10_H_13_NO_2_
*M* *_r_* = 179.21
Monoclinic, 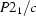

*a* = 9.2986 (19) Å
*b* = 14.713 (3) Å
*c* = 7.0551 (15) Å
β = 108.465 (8)°
*V* = 915.5 (3) Å^3^
*Z* = 4
Mo *K*α radiation
μ = 0.09 mm^−1^
*T* = 296 K
0.23 × 0.18 × 0.15 mm

#### Data collection

Bruker SMART CCD area-detector diffractometer
5022 measured reflections
1611 independent reflections
1338 reflections with *I* > 2σ(*I*)
*R*_int_ = 0.028

#### Refinement

*R*[*F* ^2^ > 2σ(*F* ^2^)] = 0.085
*wR*(*F* ^2^) = 0.277
*S* = 1.01
1611 reflections
122 parameters
H-atom parameters constrained
Δρ_max_ = 0.86 e Å^−3^
Δρ_min_ = −0.58 e Å^−3^

### 

Data collection: *SMART* (Bruker, 2004 ▶); cell refinement: *SAINT* (Bruker, 2004 ▶); data reduction: *SAINT*; program(s) used to solve structure: *SHELXS97* (Sheldrick, 2008 ▶); program(s) used to refine structure: *SHELXL97* (Sheldrick, 2008 ▶); molecular graphics: *SHELXTL* (Sheldrick, 2008 ▶); software used to prepare material for publication: *SHELXL97*.

## Supplementary Material

Crystal structure: contains datablocks global, I. DOI: 10.1107/S1600536810019951/su2178sup1.cif

Structure factors: contains datablocks I. DOI: 10.1107/S1600536810019951/su2178Isup2.hkl

Additional supplementary materials:  crystallographic information; 3D view; checkCIF report

## Figures and Tables

**Table 1:** Hydrogen-bond geometry (Å, °)

*D*—H⋯*A*	*D*—H	H⋯*A*	*D*⋯*A*	*D*—H⋯*A*
O1—H1⋯N1	0.82	1.92	2.616 (4)	142
C10—H10*B*⋯O1^i^	0.96	1.98	2.782 (4)	140

Symmetry code: (i) 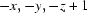.

**Fig. 1.**
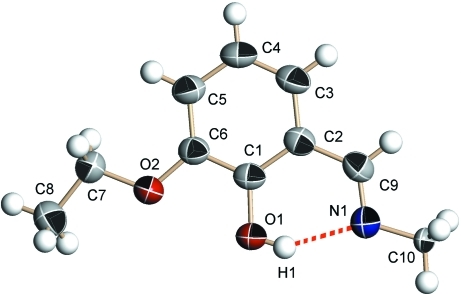
The molecular structure of the title molecule, showing 30 % probability displacement ellipsoids. The intramolecular O-H···N hydrogen bond is shown as a dashed red line.

**Fig. 2.**
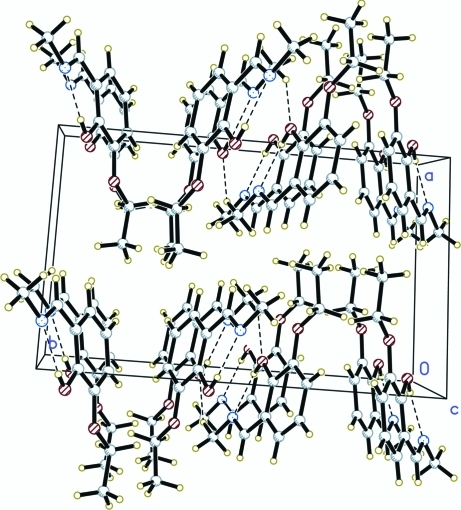
A view along the c-axis of the crystal packing of the title compound. The O-H···N and C-H···O hydrogen bonds are shown as dashed lines.

## supplementary crystallographic information

### Comment

Schiff base compounds (Zhang *et al.*, 2003; Karadayı *et al.*, 2003; Che *et al.*, 2002; Fun *et al.*, 2009; Jia *et al.*, 2009; Wang *et al.*, 2010) have aroused increasing interest because of their antiviral, anticancer and antibacterial activities. Herein, we report the synthesis and crystal structure of the new title Schiff base compound, prepared by the reaction of 2-hydrogen-3-ethoxy-benzaldehyde and methylamine.

The molecular structure of the title molecule is illustrated in Fig. 1. The bond distances and angles are similar to those found in the methoxy analogue (Chatziefthimiou *et al.*, 2006). Excluding the methyl groups (C8 and C10), all the other non-hydrogen atoms (O1/O2/N1/C1-C7/C9) lie in a plane (planar to within 0.054 (3)Å). There is an intramolecular O–H···N hydrogen bond between the phenol and imido-group (Table 1), similar to the situation in crystal structures of the methoxy analogue (Chatziefthimiou *et al.*, 2006), 6-Acetoxymethyl-3-[(2-hydroxy-3-methoxybenzylidene)-amino]-3, 4,5,6-tetrahydro-2*H*-pyran-2,4,5-triyl triacetate (Wang *et al.*, 2010) and 5,5'-Dimethoxy-2,2'-[4,5-dimethyl-*o*- phenylenebis(nitrilomethylidyne)]diphenol (Kargar *et al.*, 2010).

In the crystal molecules are linked through weak intermolecular C–H···O hydrogen bond, to form dimers centered about an inversion center (Fig. 2).

### Experimental

Compound 2-hydrogen-3-ethoxy-benzaldehyde (0.166 g, 1 mmol) was dissolved in ethanol (15 ml). To this solution was added a methylamine solution (0.5 ml) and the mixture was stirred and refluxed at 323 K for 2 h. After cooling to room temperature and filtration, the filtrate was left to stand at room temperature. Yellow block-like crystals, suitable for X-ray diffraction analysis, were obtained in a yield of 76 %. Analysis found (%): C 66.97, H 7.38, N 7.84; C_10_H_13_NO_2_ requires (%): C 67.02, H 7.31, N 7.82.

### Refinement

All the H-atoms were positioned geometrically and were treated as riding atoms: O—H 0.82 Å, C—H 0.93–0.97 Å, with *U*_iso_(H) = k × *U*_eq_(parent O or C-atom), where k = 1.2 for H-aromatic and = 1.5 for H-methyl and H-hydroxyl.

### Crystal data

**Table:**

C_10_H_13_NO_2_	*F*(000) = 384
*M**_r_* = 179.21	*D*_x_ = 1.300 Mg m^−^^3^
Monoclinic, *P*2_1_/*c*	Mo *K*α radiation, λ = 0.71073 Å
Hall symbol: -P 2ybc	Cell parameters from 1611 reflections
*a* = 9.2986 (19) Å	θ = 2.3–25.0°
*b* = 14.713 (3) Å	µ = 0.09 mm^−^^1^
*c* = 7.0551 (15) Å	*T* = 296 K
β = 108.465 (8)°	Block, yellow
*V* = 915.5 (3) Å^3^	0.23 × 0.18 × 0.15 mm
*Z* = 4	

### Data collection

**Table:**

Bruker SMART CCD area-detector diffractometer	1338 reflections with *I* > 2σ(*I*)
Radiation source: fine-focus sealed tube	*R*_int_ = 0.028
graphite	θ_max_ = 25.0°, θ_min_ = 2.3°
phi and ω scans	*h* = −10→11
5022 measured reflections	*k* = −17→17
1611 independent reflections	*l* = −8→6

### Refinement

**Table:**

Refinement on *F*^2^	Secondary atom site location: difference Fourier map
Least-squares matrix: full	Hydrogen site location: inferred from neighbouring sites
*R*[*F*^2^ > 2σ(*F*^2^)] = 0.085	H-atom parameters constrained
*wR*(*F*^2^) = 0.277	*w* = 1/[σ^2^(*F*_o_^2^) + (0.1633*P*)^2^ + 1.1252*P*] where *P* = (*F*_o_^2^ + 2*F*_c_^2^)/3
*S* = 1.01	(Δ/σ)_max_ = 0.002
1611 reflections	Δρ_max_ = 0.86 e Å^−^^3^
122 parameters	Δρ_min_ = −0.58 e Å^−^^3^
0 restraints	Extinction correction: *SHELXL97* (Sheldrick, 2008), Fc^*^=kFc[1+0.001xFc^2^λ^3^/sin(2θ)]^-1/4^
Primary atom site location: structure-invariant direct methods	Extinction coefficient: 0.024 (11)

### Special details

**Table:**

Geometry. All esds (except the esd in the dihedral angle between two l.s. planes) are estimated using the full covariance matrix. The cell esds are taken into account individually in the estimation of esds in distances, angles and torsion angles; correlations between esds in cell parameters are only used when they are defined by crystal symmetry. An approximate (isotropic) treatment of cell esds is used for estimating esds involving l.s. planes.
Refinement. Refinement of *F*^2^ against ALL reflections. The weighted *R*-factor wR and goodness of fit *S* are based on *F*^2^, conventional *R*-factors *R* are based on *F*, with *F* set to zero for negative *F*^2^. The threshold expression of *F*^2^ > σ(*F*^2^) is used only for calculating *R*-factors(gt) etc. and is not relevant to the choice of reflections for refinement. *R*-factors based on *F*^2^ are statistically about twice as large as those based on *F*, and *R*- factors based on ALL data will be even larger.

### Fractional atomic coordinates and isotropic or equivalent isotropic displacement parameters (Å^2^)

**Table:**

	*x*	*y*	*z*	*U*_iso_*/*U*_eq_	
C1	−0.0203 (4)	0.1015 (2)	0.8638 (5)	0.0465 (9)	
C2	−0.1745 (4)	0.0879 (2)	0.8267 (5)	0.0477 (9)	
C3	−0.2406 (4)	0.1178 (3)	0.9696 (6)	0.0592 (10)	
H3	−0.3434	0.1084	0.9482	0.071*	
C4	−0.1544 (5)	0.1603 (3)	1.1393 (6)	0.0654 (11)	
H4	−0.2000	0.1813	1.2308	0.078*	
C5	0.0000 (5)	0.1728 (3)	1.1776 (6)	0.0578 (10)	
H5	0.0577	0.2010	1.2952	0.069*	
C6	0.0682 (4)	0.1432 (2)	1.0401 (5)	0.0481 (9)	
C7	0.3157 (4)	0.1927 (3)	1.2361 (6)	0.0579 (10)	
H7A	0.3057	0.1632	1.3542	0.069*	
H7B	0.2889	0.2563	1.2391	0.069*	
C8	0.4756 (5)	0.1841 (4)	1.2314 (7)	0.0727 (12)	
H8A	0.4942	0.1223	1.2015	0.109*	
H8B	0.5451	0.2007	1.3592	0.109*	
H8C	0.4897	0.2236	1.1304	0.109*	
C9	−0.2684 (4)	0.0421 (3)	0.6471 (5)	0.0535 (9)	
H9	−0.3714	0.0343	0.6271	0.064*	
C10	−0.3148 (3)	−0.0314 (2)	0.3564 (4)	0.0407 (8)	
H10A	−0.3663	−0.0781	0.4052	0.061*	
H10B	−0.2616	−0.0583	0.2741	0.061*	
H10C	−0.3875	0.0116	0.2792	0.061*	
N1	−0.2115 (3)	0.0129 (2)	0.5180 (5)	0.0556 (9)	
O1	0.0527 (3)	0.0762 (2)	0.7326 (4)	0.0612 (9)	
H1	−0.0096	0.0596	0.6275	0.092*	
O2	0.2187 (3)	0.15026 (19)	1.0599 (4)	0.0590 (8)	

### Atomic displacement parameters (Å^2^)

**Table:**

	*U*^11^	*U*^22^	*U*^33^	*U*^12^	*U*^13^	*U*^23^
C1	0.0514 (19)	0.0426 (17)	0.0509 (19)	0.0052 (14)	0.0240 (15)	0.0028 (14)
C2	0.0478 (19)	0.0433 (17)	0.054 (2)	0.0058 (14)	0.0193 (15)	0.0042 (14)
C3	0.052 (2)	0.063 (2)	0.070 (2)	0.0072 (17)	0.0295 (18)	−0.0009 (19)
C4	0.065 (2)	0.075 (3)	0.067 (2)	0.0070 (19)	0.036 (2)	−0.013 (2)
C5	0.063 (2)	0.058 (2)	0.057 (2)	0.0007 (17)	0.0261 (18)	−0.0085 (17)
C6	0.0512 (19)	0.0442 (17)	0.0526 (19)	0.0023 (14)	0.0215 (16)	−0.0013 (14)
C7	0.057 (2)	0.061 (2)	0.056 (2)	−0.0070 (17)	0.0180 (17)	−0.0089 (17)
C8	0.055 (2)	0.091 (3)	0.070 (3)	−0.003 (2)	0.018 (2)	−0.005 (2)
C9	0.0439 (18)	0.059 (2)	0.058 (2)	0.0032 (15)	0.0174 (16)	0.0038 (17)
C10	0.0279 (14)	0.0597 (19)	0.0317 (15)	−0.0057 (12)	0.0057 (11)	−0.0128 (13)
N1	0.0500 (17)	0.0607 (19)	0.0542 (18)	−0.0019 (13)	0.0140 (15)	−0.0045 (14)
O1	0.0486 (15)	0.0812 (19)	0.0581 (16)	−0.0025 (13)	0.0229 (12)	−0.0191 (13)
O2	0.0493 (15)	0.0723 (17)	0.0597 (16)	−0.0071 (12)	0.0233 (12)	−0.0153 (12)

### Geometric parameters (Å, °)

**Table:**

C1—O1	1.362 (4)	C7—C8	1.503 (6)
C1—C2	1.388 (5)	C7—H7A	0.9700
C1—C6	1.398 (5)	C7—H7B	0.9700
C2—C3	1.406 (5)	C8—H8A	0.9600
C2—C9	1.457 (5)	C8—H8B	0.9600
C3—C4	1.364 (6)	C8—H8C	0.9600
C3—H3	0.9300	C9—N1	1.264 (5)
C4—C5	1.387 (6)	C9—H9	0.9300
C4—H4	0.9300	C10—N1	1.398 (4)
C5—C6	1.386 (5)	C10—H10A	0.9600
C5—H5	0.9300	C10—H10B	0.9600
C6—O2	1.366 (4)	C10—H10C	0.9600
C7—O2	1.428 (4)	O1—H1	0.8200
			
O1—C1—C2	122.7 (3)	O2—C7—H7B	110.2
O1—C1—C6	116.4 (3)	C8—C7—H7B	110.2
C2—C1—C6	120.8 (3)	H7A—C7—H7B	108.5
C1—C2—C3	118.7 (3)	C7—C8—H8A	109.5
C1—C2—C9	121.9 (3)	C7—C8—H8B	109.5
C3—C2—C9	119.4 (3)	H8A—C8—H8B	109.5
C4—C3—C2	120.3 (3)	C7—C8—H8C	109.5
C4—C3—H3	119.9	H8A—C8—H8C	109.5
C2—C3—H3	119.9	H8B—C8—H8C	109.5
C3—C4—C5	121.1 (3)	N1—C9—C2	120.8 (3)
C3—C4—H4	119.5	N1—C9—H9	119.6
C5—C4—H4	119.5	C2—C9—H9	119.6
C6—C5—C4	119.8 (4)	N1—C10—H10A	109.5
C6—C5—H5	120.1	N1—C10—H10B	109.5
C4—C5—H5	120.1	H10A—C10—H10B	109.5
O2—C6—C5	125.8 (3)	N1—C10—H10C	109.5
O2—C6—C1	114.8 (3)	H10A—C10—H10C	109.5
C5—C6—C1	119.3 (3)	H10B—C10—H10C	109.5
O2—C7—C8	107.5 (3)	C9—N1—C10	114.2 (3)
O2—C7—H7A	110.2	C1—O1—H1	109.5
C8—C7—H7A	110.2	C6—O2—C7	117.8 (3)

### Hydrogen-bond geometry (Å, °)

**Table:**

*D*—H···*A*	*D*—H	H···*A*	*D*···*A*	*D*—H···*A*
O1—H1···N1	0.82	1.92	2.616 (4)	142
C10—H10B···O1^i^	0.96	1.98	2.782 (4)	140

Symmetry codes: (i) −*x*, −*y*, −*z*+1.

## References

[bb1] Bruker (2004). *SMART* and *SAINT* Bruker AXS Inc., Madison, Wisconsin, USA.

[bb2] Chatziefthimiou, S. D., Lazarou, Y. G., Hadjoudis, E., Dziembowska, T. & Mavridis, I. M. (2006). *J. Phys. Chem. B*, pp. 23701–23709. 10.1021/jp064110p 17125330

[bb3] Che, C.-M., Kwong, H.-L., Chu, W.-C., Cheung, K.-F., Lee, W.-S., Yu, H.-S., Yeung, C.-T. & Cheung, K.-K. (2002). *Eur. J. Inorg. Chem.* pp. 1456–1463.

[bb4] Fun, H.-K., Kia, R., Kargar, H. & Jamshidvand, A. (2009). *Acta Cryst.* E**65**, o722–o723. 10.1107/S1600536809008137 PMC2968989 21582458

[bb5] Jia, Z. (2009). *Acta Cryst.* E**65**, o646. 10.1107/S1600536809003328 PMC2968515 21582294

[bb6] Karadayı, N., Gözüyeşil, S., Güzel, B., Kazak, Canan & Büyükgüngör, O. (2003). *Acta Cryst.* E**59**, o851–o853.

[bb7] Kargar, H., Kia, R., Khan, I. U., Sahraei, A. & Aberoomand Azar, P. (2010). *Acta Cryst* E**66**, o728. 10.1107/S1600536810007282 PMC2983852 21580575

[bb8] Sheldrick, G. M. (2008). *Acta Cryst.* A**64**, 112–122. 10.1107/S0108767307043930 18156677

[bb9] Wang, Y. F., Zhang, S.-H., Chen, Z. F. & Liang, H. (2010). *Acta Cryst.* E**66**, o990. 10.1107/S1600536810011475 PMC2983953 21580786

[bb10] Zhang, S. H., Jiang, Y. M., Xiao, Y. & Zhou, Z. Y. (2003). *Chin. J. Inorg. Chem.* **19**, 517–520.

